# The Korean medicine for aging cohort (KoMAC) study: A protocol for a prospective, multicenter cohort study on healthy aging in the population entering old age in South Korea

**DOI:** 10.1371/journal.pone.0316986

**Published:** 2025-02-11

**Authors:** Mi Mi Ko, Seojae Jeon, Wonbae Ha, Young-Eun Kim, So Young Jung, Bo-Young Kim, Myunghwa Kim, Kwang-Ho Choi, Geonhui Kang, So Min Lee, You Mee Ahn, Nahyun Cho, Hanbit Jin, Jungtae Leem, Seungkwan Choi, Jungho Jo, Junghan Lee, Jeeyoun Jung

**Affiliations:** 1 Korea Medicine Science Research Division, Korea Institute of Oriental Medicine, Daejeon, Republic of Korea; 2 Korea Institute of Integrative Medicine, Jangheung, Republic of Korea; 3 Department of Korean Medicine Rehabilitation, College of Korean Medicine, Wonkwang University, Iksan, Republic of Korea; 4 Korea Medicine Data Research Division, Korea Institute of Oriental Medicine, Daejeon , Republic of Korea; 5 Clinical Research Coordinating Team, Korea Institute of Oriental Medicine, Daejeon , Republic of Korea; 6 Department of Diagnostics, College of Korean Medicine, Wonkwang University, Iksan, Republic of Korea; 7 Department of Internal medicine, College of Korean Medicine, Wonkwang Universty, Iksan, Republic of Korea; 8 Research Center of Traditional Korean Medicine, Wonkwang University, Iksan, Republic of Korea; International University of Health and Welfare, School of Medicine, JAPAN

## Abstract

**Background:**

South Korea is anticipated to enter a super-aged society by 2025, necessitating a focus on healthy aging. In Korean medicine (KM), aging and disease susceptibility are individual specific, emphasizing personalized treatments, and many Korean local governments have integrated KM services for elderly people into the public sector. However, there is a notable absence of research incorporating KM to treat older adults.

**Aim:**

The proposed study aims to examine the comprehensive health profiles of individuals entering old age in rural and urban areas and explore the significant correlations between healthy aging and four key factors: biological, psychological, social, and KM-based phenotype factors. It will also establish a database and blood biobank, serving as a platform for future research to develop a traditional KM-based healthy aging model.

**Methods:**

A multiple randomized controlled trial design will be adopted in this prospective, multicenter cohort study for the clinical investigation of the markers associated with KM-based healthy aging. The aim is to recruit 1,000 participants who are entering old age from both urban and rural settings for this study, and recruitment began in August 2023 with follow-up surveys planned at one-year intervals. Comprehensive health profiles, including biological, psychological, social, and KM-based phenotype factors, will be developed through the creation of a database, a blood biobank, and multi-omics data.

**Results:**

In the baseline phase of this study, we will focus on identifying markers for KM-based phenotypes and examining how these phenotypes relate to aging and associated diseases. In the next phase, we will implement interventions tailored to KM-based phenotypes to verify the effects of KM on healthy aging. Ultimately, we intend to develop a KM-based integrated health management model, with further substudies aiming to explore factors related to healthy aging. This protocol was approved by the institutional review board of Wonkwang University Korean Medicine Hospital, Iksan, Republic of Korea (approval number: WKUIOMH-IRB-2023-05) on August 16, 2023 and Jangheung Integrative Medical Hospital (approval number: WKUJIM-202307-001) on August 21, 2023. Recruitment started on August 16, 2023.

**Conclusion:**

The anticipated results of our study aim to establish personalized preventive and therapeutic interventions for individuals entering old age. Additionally, we seek to offer an KM-based integrated health management model that incorporates comprehensive diagnosis and an integrative medical treatment strategy for healthy aging.

**Trial Registration:**

Clinical Research Information Service: KCT0008863 (registered on October 11, 2023, https://cris.nih.go.kr/cris/search/detailSearch.do/25718).

## Introduction

According to Statistics Korea, South Korea is anticipated to transition into a super-aged society by 2025, just seven years after entering an aged society in 2018. By the year 2060, it is estimated that 43.9% of the population will be classified as elderly [1]. As the super-aged era approaches, there is a growing societal emphasis on enhancing quality of life and placing greater attention on promoting healthy aging to ensure that individuals age while maintaining a state of well-being [2].

The World Health Organization (WHO) defines healthy aging as a holistic approach that prioritizes intrinsic capacity, functional ability, and well-being, with an emphasis on an inclusive environment, integrated care, community support, and chronic disease management for a positive aging experience [2,3]. With the increasing interest and demand for healthy aging, the focus has been on improving quality of life through integrative medical treatments through multidisciplinary diagnoses and preventive screenings, as opposed to simple therapies [4,5].

As part of these initiatives, various studies are underway to propose strategies for healthy aging [5]. Notably, significant studies, including the Korean Longitudinal Study of Aging, have focused on aging and the quality of life of elderly individuals in South Korea [6]. Additionally, a Korean frailty and aging cohort study concentrated on physical health and chronic conditions among Korean seniors [7]. Moreover, the Korean Brain Aging Study for Early Diagnosis and Prediction of Alzheimer’s Disease was conducted, focusing on brain cognitive functions, aging, and Alzheimer’s disease [8]. Despite numerous cohort studies targeting Koreans, these efforts have yet to incorporate assessment criteria related to traditional Korean medicine (KM). Additionally, there is a notable absence of cohort studies presenting health management methods that incorporate the concepts of traditional KM in the context of healthy aging among elderly people [9].

In particular, in KM, the aging process and susceptibility to disease vary according to an individual’s phenotype, and KM emphasizes personalized treatments and preventive measures tailored to an individual’s unique characteristics [10]. Thus, KM continues to be widely utilized among many Koreans, coexisting seamlessly with Western medicine. Additionally, the Korean government has incorporated KM services for elderly individuals into the public sector, and 242 local governments (46.5% of localities) provide public TKM services [11]. This underscores the need for studies within cohorts that include KM. In addition, South Korea experiences differences in the living environments of elderly people in rural and urban areas, as well as variations in community cohesion and health behaviors among residents. Many studies have compared these characteristics and investigated life satisfaction [12–14]. Therefore, in the context of South Korea’s elderly cohort studies, there is a need for research that not only targets the general population but also considers the regional characteristics of the participants.

In this study, we aim to examine the comprehensive health profiles of individuals entering old age in rural and urban areas and explore the significant correlations between healthy aging and four key factors: biological, psychological, social, and KM-based phenotype factors. Additionally, we will establish a database and blood biobank. Furthermore, this study will serve as a recruitment platform for future intervention studies using a cohort multiple randomized controlled trial design [15], contributing to the development of a health management model for promoting healthy aging based on traditional KM. Ultimately, we intend to develop a traditional KM-based healthy aging model, with further substudies aiming to explore factors related to healthy aging based on KM-based phenotypes.

## Methods

### Study design

**Fig 1:**
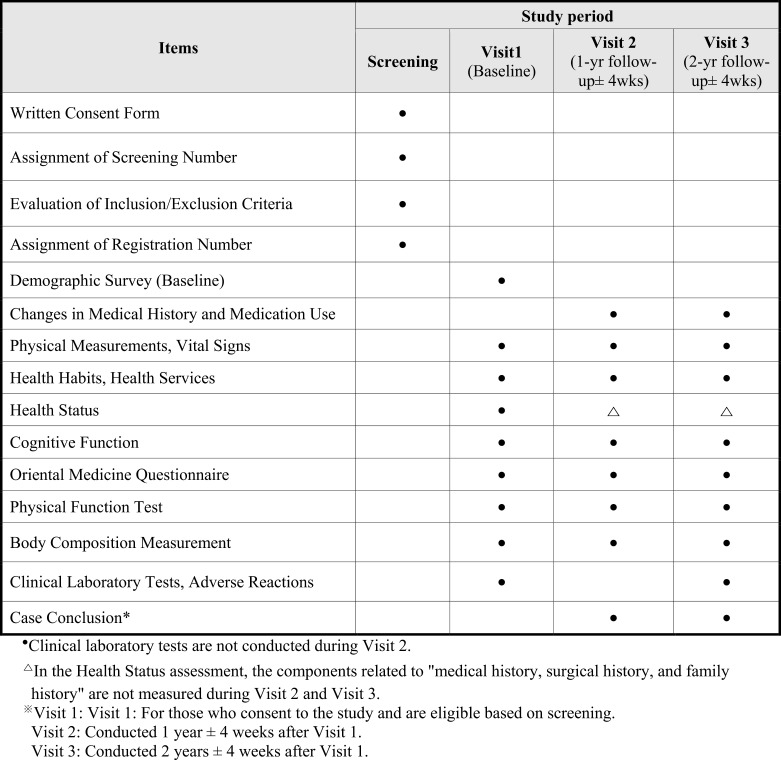
Schedule of assessments and procedures for aging cohort (KoMAC) study. Screening: Initial assessment phase before baseline visit, including consent and eligibility checks. Visit 1 (Baseline): Baseline data collection, including demographic survey, physical measurements, health habits, and initial health assessments. Visit 2 (1-year follow-up ±  4 weeks): Follow-up assessments conducted 1 year after baseline to monitor health changes, excluding clinical lab tests. Visit 3 (2-year follow-up ±  4 weeks): Final follow-up assessments conducted 2 years after baseline, similar in scope to Visit 2.

This will be a prospective, multicenter cohort study that adopts a multiple randomized controlled trial design [15] to clinically investigate markers associated with KM-based healthy aging. The baseline phase is scheduled from August 2023 to December 2024, aiming to recruit 1,000 participants. Each participant will undergo two follow-up assessments, the first occurring one year post-enrollment, extending the study period through 2025 (Fig 1).

### Study sample

This study will be conducted with a target group of 1000 participants who are entering old age and who are recruited from Wonkwang University Korean Medicine Hospital, a medium-sized city, and Jangheung Integrative Medical Hospital, a rural area in South Korea. The objective is to investigate potential cohort losses due to yearly mortality and other factors, with a target panel retention rate exceeding 80%. Despite limitations in achieving perfect representativeness of the population, practical feasibility will be considered by selecting research participants based on the demographic distribution of the population aged 50–65 years in Iksan and Jangheung, Jeonbuk, as of 2023 [16,17]. Considering the budget for this cohort project and the target population of individuals entering old age at Iksan and Jangheung in 2023 (for a total of 82,498 individuals), the goal is to recruit approximately 1% of this target population while factoring in a panel dropout rate of 20%. Therefore, the aim is to register a total of 1,000 adult participants, encompassing both sexes.

### Study population

This study will be This proposal involves recruiting 1,000 individuals over a two-year period from August 2023 to December 2024 for the study of individuals entering old age. Eligible participants will be identified by Clinical Research Coordinators (CRCs) from each institution who receive thorough training regarding the inclusion/exclusion criteria and recruitment procedures.

The inclusion criteria are as follows: (1) adults aged 50 to 65 years; (2) adults residing in either a medium-sized city (Iksan city and nearby areas in Jeonbuk state) or a rural area (Jangheung County and nearby areas in Jeonnam state); (3) adults who can read and respond to the questionnaire; and (4) adults who understand the purpose of the study and provide written consent to participate. The exclusion criteria are as follows: (1) a history of major psychiatric disorders diagnosed by the DSM-5, such as psychotic symptoms, schizophrenia spectrum disorders, delusional disorders, bipolar disorders, alcohol or substance use disorders; (2) the inability to communicate effectively or limitations in reading and writing; and (3) determination by the research team or responsible personnel that the study cannot be completed due to a medical condition. Additionally, (1) participants for whom the selected or excluded criteria are violated following screening, (2) participants who withdraw consent during the study, and (3) participants who are not traceable will be considered premature withdrawals from this study.

After registration, follow-up examinations will be performed at 1-year intervals from August 2024 to December 2025. However, blood tests will be administered every two years: at baseline and during the final follow-up. A strategy for sustained participation will be adopted to keep participants engaged in the cohort study. This will include sending quarterly health management information through a webzine and providing free health consultation services to prevent dropout of participants. Additionally, substantial compensation will be provided every visit, along with immediate explanations of the health screening process, to minimize participant attrition.

### Study procedures

After commencing the research, banners for participant recruitment will be displayed in local public health care institutions and health centers. Contact will be initiated with individuals who respond to the banners; their age will be verified and visit schedules will be coordinated. Participants will be asked to maintain fasting for eight hours before their scheduled institution visit and to bring photographs of any nutritional supplements or medications that they are currently taking.

At the first visit, a research nurse will provide an overall explanation of the study. Then, the specific tests scheduled for the day will be detailed using a worksheet, with the nurse providing thorough explanations and addressing any queries immediately. After completing the explanations, participants will sign the research consent form and the consent form for the use of human-derived materials.

A medical registration form will be provided only to participants who sign both forms. For fasting patients, blood tests will be conducted first, and a light snack will be provided for those who complete the blood draw. Subsequently, participants will complete the all surveys and examinations. The entire examination process will take approximately 1 hour and 30 minutes. Upon completion, participants will be provided with information regarding their next examination date. Recruitment began on August 16, 2023, at Wonkwang University Korean Medicine Hospital and on August 22, 2023, at Jangheung Integrative Medical Hospital. It is expected to be completed by December 31, 2025.

### Outcome measures

Various outcomes will be examined to assess the health profiles of individuals entering old age in rural and urban areas and to investigate the meaningful associations between healthy aging and the following four factors: biological factors, psychological factors, social factors, and KM-based phenotype factors [18–38]. Biological factors include sex, age, weight, height, BMI, blood pressure, pulse rate, body temperature, overall wellness, smoking and alcohol history, physical activity, nutritional status, dietary habits, appetite, supplement intake, constipation degree, comorbidities, daily living activities, physical resilience, oral and gynecological health, medical, surgical, and medication histories, family medical history, physical performance, knee and lumbar joint range of motion, core and grip strength, hearing, vision, pulmonary function, pulsometry, brain wave examination, bone density, body composition, and clinical lab tests, including biochemical, hormonal, hematological markers, urine tests, and omics profiles (metabolomics/lipidomics, proteomics). Psychological factors include assessments of overall wellness, sleep quality, quality of life, fear of falling, depression levels, and cognitive function. Social factors include education level, family and social relationships, pregnancy and lactation history, overall wellness, smoking and alcohol history, healthcare access, and quality of life. KM-based phenotype factors such as kidney deficiency, five organ deficiency, blood stasis, and core seven-emotions status will also be evaluated. Table 1 provides details of the measurements and instruments used in this study to be acquired.

**Table 1:** Measurement and instruments used in the Korean Medicine for Aging Cohort (KoMAC) study.

Category	Variables	Instruments and measurement	Visit
1. Sociodemographic	Sex, age, education level, family relationships, pregnancy and lactation history	Each factor will be collected using self-report.	1,2,3
2. Anthropometric parameters	Weight, height, body mass index	Each factor will be measured by BSM330. (InBody Co., Ltd., Korea)	1,2,3
3. Vital signs	Blood pressure, pulse rate, body temperature	Blood pressure, pulse rate will be measured by BPBIO320, InBody Co., Ltd., Korea) and body temperature will be determined by Non-contact forehead thermometer HFS-900. (HuBDIC Co., Ltd., Korea).	1,2,3
4. Health habits	Health habit about following 8 items will be measured; a. Overall wellness status b. Smoking and alcohol consumption history c. Quality of sleep d. Physical activity e. Nutritional status f. Dietary survey g. Appetite h. Social relationships	a. The Paving the Path to wellness questionnaire. b. The factor will be collected using self-report. c. Korean version of the Pittsburgh Sleep Quality Index, a subjective assessment of sleep quality over the past month, comprising 7 domains with a total of 18 questions. Scores range from 0 to 3, with higher scores indicating lower sleep quality [18]. d. The Korean Short Form of the International Physical Activity Questionnaire (IPAQ) records moderate-intensity physical activity, high-intensity physical activity, and walking performed over the past 7 days. The IPAQ score conversion guidelines are used to convert physical activity time into Metabolic Equivalent of Task (MET-min/week) scores [19]. e. The Mini Nutritional Assessment Short Form comprises 6 questions. With a total score of 14 points, a score of 12–14 indicates normal nutritional status, 8–11 suggests a risk of malnutrition, and a score of 0–7 signifies malnutrition [20]. f. To assess the overall quality of the participants’ diet, variables such as the frequency of dining out per week, companions during meals, nutritional education, and awareness of nutritional labeling are used in the dietary survey [21]. g. The Simplified Nutritional Appetite Questionnaire comprises 4 items rated on a 5-point scale, where lower scores indicate poor appetite status [22]. h. The Medical Outcomes Study-Social Support Survey is a questionnaire used to assess the nature and strength of social support among participants. It consists of a total of 19 questions rated on a 5-point scale, where higher scores indicate higher levels of social support [23].	1,2,3
5. Health service status	Accessibility of Healthcare Services	Unmet medical needs refer to the experience where individuals responded “yes” to the question, “In the past year, have there been times when you wanted to visit a hospital or clinic (excluding dental care) but could not?” This defines instances of unmet medical services (excluding dental care) as unmet medical needs [24].	1,2,3
6. Health condition	Following biological and psychological factors of health condition will be determined every year; a. Quality of life b. Intake of health supplements and symptom relief, personal satisfaction c. Degree of constipation d. Degree of depression e. Fear of falling f. Comorbidities g. Activities of Daily Living h. Physical resilience (The Physical Resilience Scale) i. Oral health status, gynecological history, medical history, surgical history, medication history and family medical history	a. The SF-12 comprises two domains: the Physical Component Score and the Mental Component Score. It consists of a total of 8 questions rated on a 5-point scale, where higher scores indicate better health [25]. The EuroQol Group developed the EuroQOL 5-Dimension 5-level, which includes the EQ-5D assessing health status across 5 dimensions, and the EQ-5D Visual Analog Scale questioning subjective health levels. Higher scores signify higher quality of life [26]. b. The self-report questionnaire will be used to assess the health supplement use and satisfaction of participants in this study. Participants will be asked to indicate whether they currently use any health supplements, and if so, and how satisfied they are with the effects of these supplements. c. The Constipation Assessment Scale developed by McMillan and Williams (1989) comprises 8 items rated on a 3-point scale. Higher scores indicate more severe constipation [27]. d. The Geriatric Depression Scale Short Form Korean Version is a 15-item shortened depression assessment tool. It considers a cutoff score of 8 points or higher to determine the presence of depression [28]. e. Korean Falls Efficacy Scale-International consists of 16 items, rated on a scale of 0 to 64, where a higher score indicates lower self-efficacy [29]. f. The Charlson Comorbidity Index (CCI) is based on a study conducted in 1984 at the New York Hospital, where the medical records of 604 patients hospitalized for one month were analyzed. It identified 19 conditions that predicted one-year mortality. Each condition is assigned a weighted score (1, 2, 3, 6 points) based on the relative risk of each illness. The sum of these weights comprises the CCI [30]. g. The Korean version of the Instrumental Activities of Daily Living comprises 10 areas, with each item scored from 3 to 4 points, totaling 37 points. Higher scores indicate greater functional impairment, suggesting an increased need for care [31]. h. The Physical Resilience Scale is a tool focusing on recovery related to illness, comprising 15 items. Scores range from 0 to 15, where higher scores indicate a higher level of resilience in recovery from illness [32]. i. Each factor will be collected using self-report.	1,2,3
7. Cognitive function	Cognitive function	Cognitive function will be determined by the Korean version of the Montreal Cognitive Assessment [33].	1,2,3
8. traditional KM questionnaire	The KM-based phenotype will be assessed by following questionnaire; a. Kidney deficiency b. Five Organ deficiency c. Blood stasis status d. The Core Seven-Emotions status	a. Kidney deficiency pattern assessment questionnaire, this questionnaire designed for this study will be employed to assess its validity [34]. b. Assessment using the Instrument of Korean Medical Pattern Identification and Functional Evaluation for Five Organ [35] c. Assessment using the Blood Stasis Questionnaires on metabolic syndrome [36] d. Assessment using the Core Seven-Emotions Inventory Short Form [37]	1,2,3
9. Physical function test	Physical function will be collected by following assessment; a. Physical performance b. Range of Motion for knee and lumbar joints assessment c. Core-muscle strength d. Grip strength e. Hearing f. Vision g. Pulmonary function h. Brain wave and pulsometry	a. The Short Physical Performance Battery consists of three components: balance tests (side-by-side stand, semi-tandem stand, tandem stand), gait speed (4-meter walk), and chair stands (5 repetitions). Each section is scored out of 4 points, contributing to a total score of 12 points [38]. b. To assess the range of motion of the knee joint, the flexion and extension of the knee joint are measured using a goniometer. To assess the range of motion of the lumbar spine, the flexion and extension of the lumbar spine are measured using an inclinometer. c. To perform McGill’s core endurance test, the following tests are performed sequentially: isometric endurance test, flexor endurance test, extensor endurance test, and side bridge test. d. After confirming the dominant hand, the left and right grip strength is measured two set, three times each, at three-minute intervals, using the TKK-5401 (TAKEI Scientific Instruments Co.,Ltd., Japan). e. Using the GSI-18 (GRASON-STADLER INC., America), the minimum audible range of hearing in the left and right ear is evaluated by presenting sounds from 1000Hz to 4000Hz. f. The vision test will use the Snellen eye chart to examine the left and right vision. g. Pulmonary function tests were conducted using a Spirometer SB80B (Contec Medical Systems Co., Ltd., China). h. Brain waves and pulsometry will be measured using OMNIFIT Mindcare medical (BICO CO., LTD., Korea), and HRV, brain score, brain activity, brain stress, brain imbalance, and concentration will be evaluated.	1,2,3
10. Body composition measurements	Bone density, body composition	Bone density and body composition is will be measured by using QDR 2000 Densitometer Machine (Hologic Inc., America) and Inbody 770 (InBody Co., Ltd., Korea), respectively.	1,2,3
11. Clinical laboratory	• Blood test a. Biochemistry b. Hormone c. Hematology • Urine test • Omics test e. Metabolomics/lipidomics f. Proteomics	The blood test will be performed using a QDR 2000 densitometer machine (Hologic Inc., America). a. The biochemistry will be used to check the levels of Albumin, AST, TP, Total bilirubin, Ca, Cl, Pi, Na, ALP, Amylase, CK, ALT, LDH, γ-GTP, BUN, Creatinine, Uric acid, Total cholesterol, HDL cholesterol, Triglyceride, LDL cholesterol, and Glucose through blood analysis. b. The hormones to be checked are 25-OH Vitamin D, Osteocalcin, Cortisol, Free T4, TSH, DHEA-S, Vitamin B12, Insulin, GDF8, GDF15, Testosterone, PSA, FSH, LH, and Estradiol. c. The hematology test will check the following: white blood cells, red blood cells, hemoglobin, hematocrit, platelets, mean corpuscular volume, mean corpuscular hemoglobin, mean corpuscular hemoglobin concentration, neutrophils, lymphocytes, monocytes, eosinophils, basophils, and glycated hemoglobin. d. We will be checking the following items in a urine test: Urine WBC, Specific gravity, pH, Protein, Glucose, Occult Blood, Ketones, Urobilinogen, Bilirubin, Nitrite, WBC, RBC, Epithelial cell, Bacteria, Casts, Crystal, Other e. GC/LC-MS based untargeted metabolomics analysis will be conducted to profile the comprehensive metabolic data from blood of participants f. Beads-based multiplex immunoassay will be executed to acquire the proteomics data of blood	1,3

Biological factors: sex, age, weight, height, body mass index, blood pressure, pulse rate, body temperature, overall wellness status, smoking and alcohol consumption history, physical activity, nutritional status, dietary survey, appetite, intake of health supplements, degree of constipation, comorbidities, activities of daily living, physical resilience, oral health status, gynecological history, medical history, surgical history, medication history and family medical history, physical performance test, range of motion assessment for the knee and lumbar joints, core muscle strength measurement, grip strength measurement, hearing, vision, pulmonary function, pulsometry, brain wave examination, bone density, body composition, clinical laboratory examination (biochemical markers, hormones, hematological markers, urine test), and omics profiles (metabolomics/lipidomics, proteomics).

Psychological factors: overall wellness status, quality of sleep, quality of life, fear of falling, degree of depression, and cognitive function. Social factors: education level, family relationships, pregnancy and lactation history, overall wellness status, smoking and alcohol consumption history, social relationships, accessibility of health care Services, and quality of life. KM-based phenotype factors: kidney deficiency, five organ deficiency, blood stasis, and core seven-emotions status.

### Data management and monitoring

All the data collected in this cohort study will be reported in an electronic case report form and managed on a web-based platform (iCReaT; Korea National Institute of Health, Cheongju, Republic of Korea). CRCs will be trained in using this system before initiating data entry and will enter the data according to standardized data entry guidelines. The data management will be coordinated by the Korea Institute of Oriental Medicine. Initially, a monitoring agent will be dispatched to each institution once per week for the first month, followed by monthly visits to ensure a seamless research process.

### Statistical analysis

We will conduct various analyses within the cohort. The results of the participants’ demographic data, the questionnaire-based prevalence of age-related symptoms and KM-based phenotype, physical function, and other outcome variables will be summarized using descriptive summary measures. For continuous outcomes, both univariate and multivariate linear regression analyses will be employed, while logistic regression will be used for dichotomous outcomes. Specifically, group differences (between KM-based phenotypes or between rural and urban areas) will be analyzed using Student’s t test and analysis of variance. The relationships between KM-based phenotypes and physical and psychological health, as well as demographic variables, will be analyzed using multiple regression.

Additionally, missing data patterns will be examined, and appropriate methods for handling missing data, such as Last Observation Carried Forward (LOCF) or Multiple Imputation, will be applied. Mixture models will be utilized to identify subgroups of subjects based on their biological and psychological characteristics. To estimate the cumulative risk of outcomes, Kaplan‒Meier analysis will be employed. Adjustments for covariates will be made before the follow-up period, during which Cox proportional hazard models will be used to estimate the risk of experiencing any of the outcomes. In addition, prediction modeling will be attempted by incorporating new covariates under investigation. P values less than 0.05 will be considered to indicate statistical significance. All statistical analyses will be conducted using SAS® version 9.4.

### Patient and public involvement

Patients and/or the public were not involved in this research’s design, conduct, reporting, or dissemination plans as it describes a protocol.

### Ethics and dissemination

This study received approval from the Institutional Review Board (IRB) of Wonkwang University Korean Medicine Hospital, Iksan, Republic of Korea (approval number: WKUIOMH-IRB-2023-05) on August 16, 2023 and Jangheung Integrative Medical Hospital (approval number: WKUJIM-202307-001) on August 21, 2023. Written informed consent will be obtained from all participants prior to their enrollment in the study. This study is registered with the Clinical Research Information Service (https://cris.nih.go.kr/cris/en/) (KCT0008863), and the current protocol version is 1.3. The results from this cohort study will be disseminated through the publication of peer-reviewed manuscripts, presentations at scientific meetings, and/or conferences.

## Results

In the baseline phase of this study, we will focus on identifying markers for KM-based phenotypes and examining how these phenotypes relate to aging and associated diseases. In the next phase, we will implement interventions tailored to KM-based phenotypes to verify the effects of KM on healthy aging. Ultimately, we intend to develop a KM-based integrated health management model, with further substudies aiming to explore factors related to healthy aging.

## Discussion

South Korea is currently navigating the challenges of transitioning into a super-aged society, emphasizing the critical importance of proactive health management for healthy aging before individuals reach the age of 65 years [1–5]. This paper outlines a multifaceted research protocol aimed at revealing the complexities associated with aging in both urban and rural settings, considering the distinctive characteristics that are prevalent in various regions of Korea. Data collection for this study began in August 2023 and is being carried out in two phases. Phase I comprises a cross-sectional cohort study (baseline) detailed in this paper. Participants who are included in the cohort will be given the option of allowing their data to be used for analyses and identifying them for future research interventions or for comparison purposes for intervention trials. Details of the phase II interventions will be published in full manuscripts separately.

In particular, a key aspect of our study involves analyzing KM-based phenotypes to examine aging patterns and certain diseases associated with each phenotype and evaluating the KM-based integrated health management model. To achieve this goal, in phase I, our initial focus is on identifying diagnostic markers that enable the application of KM by conducting multi-omics analyses, including hormone, immune, metabolite markers. We will subsequently explore age-related symptoms or vulnerable diseases associated with KM phenotypes through cohort studies. In phase II, we plan to conduct multiple randomized controlled trials that apply interventions such as community programs specifically tailored to well-defined KM-based phenotypes identified in phase I to verify the effects of KM on healthy aging. Based on these results, we intend to develop an integrated health management model for healthy aging, and establish a comprehensive database and blood biobank of population entering old age in rural and urban areas of Korea.

In addition, this study will include various physical function assessments, such as range of motion assessments for the knee and lumbar joints, McGill’s core endurance test, bone density tests and bioelectric impedance analysis. These assessments are expected to provide information on muscle atrophy, osteoporosis, and core muscle weakness, which are currently discussed in relation to health issues and functional decline associated with aging [39,40]. Additionally, mental health assessments and EEG analysis will be used to evaluate cognitive and emotional functions, offering insights into psychological changes associated with aging. The study will also explore the connection between physical and psychological decline and various blood markers, including multi-omics data, to provide a comprehensive understanding of age-related health deterioration.

We have established the target number of participants for this cohort at 1,000. Although this number has limitations in representing the entire population of old age, it is equivalent to 1% of the target population, based on the demographic distribution of medium-sized cities and rural areas of South Korea, taking into account the duration and scope of this project. Despite the relatively modest sample size, the study’s strength lies in its potential to reveal connections between KM-based phenotypes and aging patterns. It also aims to provide an integrated health management model that incorporates thorough diagnosis and an integrative medical treatment strategy.

## Supporting information

S1 Text Checklist. (PDF)

S2 Text KOMAC protocol. (PDF)

## Abbreviations

CRCs: Clinical Research Coordinators
IRB: Institutional Review Board
KM: Korean medicine
KoMAC: The Korean Medicine for Aging Cohort
WHO: World Health Organization.
