# Supplementary material for: The Korean medicine for aging cohort (KoMAC) study: A protocol for a prospective, multicenter cohort study on healthy aging in the population entering old age in South Korea
Source: PLoS One. 2025 Feb 11;20(2):e0316986. doi: 10.1371/journal.pone.0316986 (PMC11813093; doi:10.1371/journal.pone.0316986)
Supplement: S2 Text — (PDF) [file pone.0316986.s002.pdf]

Korean Medicine for Aging Cohort (KOMAC): Study Protocol (ver. 1.3)

Chief Investigator: Prof. Junghan Lee

Research Team: Dr. Jeeyoun Jung, Dr. Seojae Jeon, Dr. Mi Mi Ko, Dr. Wonbae Ha, Young-Eun Kim, So Young Jung, Bo-Young Kim, Dr. Myunghwa Kim, Kwang-Ho Choi, Geonhui Kang, Dr. So, Min Lee, Dr. You Mee Ahn, Nahyun Cho, Hanbit Jin, Prof.Jungtae Leem, Prof.Seungkwan Choi, Prof. Jungho Jo

Background

1. Entering a Super-Aged Society in 2025, with 43.9% of the Population Expected to Be Elderly by 2060

- Entering a Super-Aged Society: According to Statistics Korea, South Korea will enter a 'super-aged society' in 2025, just seven years after becoming an 'aging society' in 2018. By 2060, it is projected that 43.9% of the population will be elderly.
- Rising Elderly Dependency Ratio: Due to the declining working-age population (15-64 years old), the elderly dependency ratio—which measures the number of people aged 65 or older per 100 working-age individuals—is expected to increase from 21.7 in 2020 to 51.0 by 2036 and 91.4 by 2060. To offset the financial burden of supporting those aged 75 or older, government support, including the basic pension, rose from KRW 12.834 trillion in 2010 to KRW 28.96 trillion in 2016.
- Healthcare Costs for the Elderly: In 2018, the cost of treating elderly individuals exceeded 40% of the total healthcare expenses. By 2025, the cost of treating those aged 65 or older is estimated to approach KRW 60 trillion.
- Chronic Diseases Among the Elderly: Although Korea is expected to become a super-aged society, 89.5% of the elderly suffer from chronic diseases. On average, they experience 2.7 chronic conditions, with 51.0% having three or more. The prevalence of polypharmacy is also high. This trend is likely to increase medical expenses, adding to the burden on both individual finances and national healthcare systems. The World Health Organization (WHO) has recommended that nations take responsibility for managing chronic diseases among the elderly.
- Perceived Health Status: Only 32.0% of elderly Koreans consider themselves healthy, which is less than half the OECD average and ranks among the lowest. For comparison: OECD average (67.9%), Canada (88.6%), United States (87.9%).

2. The Need for Research on Healthy Aging in a Hyper-Aged Society

- Demand for Healthy Aging: As society ages, the demand for 'healthy aging'—which involves preventing and managing aging in a healthy state—is increasing. The WHO's 'Healthy Aging 2020-2030 Plan' emphasizes this, defining healthy aging as a state characterized by low disease incidence, high cognitive and physical function, and active participation in social activities. This contrasts with 'general aging,' where aging-related diseases typically accompany the normal aging process.
- Global Growth of the Aging Research Industry: As the global population ages, research into aging is emerging as a significant growth industry. The global anti-aging and service

market is projected to grow at an average annual rate of 6.5%, from $62.5 billion in 2017 to $88.6 billion (109 trillion won) by 2022.

3. Contributions of Traditional Korea Medicine to Healthy Aging

- Traditional Medicine Approaches: Traditional medicine offers various methods for preventing and managing aging. Techniques such as acupuncture, moxibustion, and herbal medicine are used to control the body's fundamental energy, with reported effects in preventing and treating aging-related diseases. Aging causes changes in biological rhythms, such as hormone secretion, sleep patterns, body temperature maintenance, and blood pressure regulation, which can contribute to geriatric diseases and hinder healthy aging.
- Traditional Methods for Healthy Aging: *Donguibogam*, a classic text in Korea medicine, outlines various methods for healthy aging and living in harmony with nature and one's own biorhythm. These include '*Sasijeolui* (四時節宣),' '*Yeonjebeop* (煉臍法),' '*Hunjebibang* (熏臍秘方),' '*Gujebeop* (灸臍法),' and '*Taesik* (胎息).'

3. Research on Aging-Related Diseases

- Ongoing Studies in the US and Europe: As the elderly population grows, research on various aging-related diseases, including kidney, heart, and diabetes, is being actively conducted in the US and Europe.
- In Korea, the Korean Senior Citizens' Association has been conducting a cohort study on the elderly population since 2006 and is also analyzing kidney weakness. However, despite these efforts, research on biomarkers and the establishment of registries related to healthy aging in Traditional Korea Medicine remains insufficient

Aims and objectives

This study aims to examine the comprehensive health profiles of individuals entering old age in rural and urban areas and explore the significant correlations between healthy aging and four key factors: biological, psychological, social, and KM-based phenotype factors. It will also establish a database and blood biobank, serving as a platform for future research to develop a traditional KM-based healthy aging model.

Study design and methods

Study design

This will be a prospective, multicenter cohort study that adopts a multiple randomized controlled trial design to clinically investigate markers associated with KM-based healthy aging. This study of baseline phase is scheduled to be conducted over a 3-year period from 2023 to 2025. Cohort registration and baseline surveys commenced in August 2023, with follow-up surveys planned at one-year intervals.

1) Eligibility criteria

Inclusion:

The inclusion criteria are as follows:

(1) adults aged 50 to 65 years

(2) adults residing in either a medium-sized city (Iksan city and nearby areas in Jeonbuk state) or a rural area (Jangheung County and nearby areas in Jeonnam state)

(3) adults who can read and respond to the questionnaire

(4) adults who understand the purpose of the study and provide written consent to participate

Exclusion:

The exclusion criteria are as follows:

(1) a history of major psychiatric disorders diagnosed by the DSM-5, such as psychotic symptoms, schizophrenia spectrum disorders, delusional disorders, bipolar disorders, alcohol or substance use disorders

(2) the inability to communicate effectively or limitations in reading and writing

(3) determination by the research team or responsible personnel that the study cannot be completed due to a medical condition.

Additionally, participants for whom the selected or excluded criteria are violated following screening, participants who withdraw consent during the study, participants who are not traceable will be considered premature withdrawals from this study.

2) Sampling and recruitment

1) Survey Population and Target Number of Registered Participants

① Target Area:

- Medium-sized Cities: *Iksan-si, Jeonbuk,* and surrounding areas
- Rural Areas: *Jangheung-gun, Jeonnam*, and surrounding areas

② Age Group:

Target Age Group: Adults aged 50-65 years, entering the elderly stage

- Target Number of Registered Participants: 1,000 participants (competitive recruitment by institution)
- Assignment of Screening Numbers and Identification Codes: Each participant who provides written consent to join the clinical study will be assigned a unique screening number and identification code.
- Registry Establishment and Recruitment Process:
  - ✓ Registry Management: tWonkwang University Korean Medicine Hospital will oversee the establishment of the registry.
  - ✓ Recruitment Implementation:

① Recruitment will be conducted in medium-sized cities by the Wonkwang University Korean Medicine Hospital and in rural areas by the Jangheung Integrative Medical Hospital.
② Recruitment notices will be distributed with the cooperation of the Iksan City Health Center, Health Branch, Jangheung-gun Health Center, and Health Branch.
③ Recruitment notices will also be posted on the websites of each participating institution.
✓ Cohort Study Management:
① Each institution will employ a research nurse responsible for the cohort study.
② A communication system will be established to regularly monitor and ensure the success of participant recruitment.

2) Sample Extraction Method

- Survey Plan: The goal is to survey a total of 1,000 participants over two years, with 500 individuals recruited each year.
- Cohort Retention: Given the possibility of cohort loss due to deaths and other factors, the aim is to maintain a panel retention rate of 80% or higher.
- Selection Criteria: While there may be limitations in the representativeness of the sample, the selection of participants will be based on the population distribution of those aged 50-65 in Iksan and Jangheung, Jeollabuk-do, as of 2023, considering practical applicability.
- Budget and Population Considerations: With a target population of 82,498 individuals aged 50-65 in Iksan and Jangheung in 2023, and accounting for a potential 20% panel dropout rate, the goal is to register a total of 1,000 adult men and women.

Iksan-si, Jeollabuk-do				*Jangheung-gun, Jeollanam-do*			
Age (Years)	Population	Male	Female	Age (Years)	Population	Male	Female
50	4,829	2,474	2,355	50	504	250	254
51	4,717	2,413	2,304	51	513	291	222
52	4,966	2,573	2,393	52	607	341	266
53	4,782	2,387	2,395	53	565	323	242
54	5,319	2,742	2,577	54	625	345	280
55	4,579	2,256	2,323	55	594	333	261
56	4,892	2,477	2,415	56	601	334	267
57	4,787	2,458	2,329	57	681	388	293
58	4,759	2,429	2,330	58	672	350	322
59	4,161	2,112	2,049	59	591	297	294
60	5,129	2,635	2,494	60	719	406	313
61	4,601	2,337	2,264	61	666	329	337
62	5,649	2,770	2,879	62	833	430	403
63	5,116	2,536	2,580	63	772	394	378
64	4,512	2,226	2,286	64	757	385	372
Total	72,798	36,825	35,973	Total	9,700	5,196	4,504

< Population Information: 2023 Estimated Population Results - Elderly Entry Group >

[Source: 2023 Jangheung Statistical Yearbook, 2023 Age-Specific Population Statistics of Iksan City]

3) Study procedures

Item	Study period			
	**Screening**	**Visit1** (Baseline)	**Visit 2** (1-yr follow-up± 4wks)	**Visit 3** (2-yr follow-up± 4wks)
Written Consent Form	●			
Assignment of Screening Number	●			
Evaluation of Inclusion/Exclusion Criteria	●			
Assignment of Registration Number	●			
Demographic Survey (Baseline)		●		
Changes in Medical History and Medication Use			●	●
Physical Measurements, Vital Signs		●	●	●
Health Habits, Health Services		●	●	●
Health Status		●	△	△
Cognitive Function		●	●	●
Oriental Medicine Questionnaire		●	●	●
Physical Function Test		●	●	●
Body Composition Measurement		●	●	●
Clinical Laboratory Tests, Adverse Reactions		●		●
Case Conclusion*			●	●

● Clinical laboratory tests are not conducted during Visit 2.

△ In the Health Status assessment, the components related to "medical history, surgical history, and family history" are not measured during Visit 2 and Visit 3.

※ Visit 1: Visit 1: For those who consent to the study and are eligible based on screening.

※ Visit 2: Conducted 1 year ± 4 weeks after Visit 1.

※ Visit 3: Conducted 2 years ± 4 weeks after Visit 1.

4) Data collection

Various outcomes will be examined to assess the health profiles of individuals entering old age in rural and urban areas and to investigate the meaningful associations between healthy aging and the following four factors: biological factors, psychological factors, social factors, and KM-based phenotype factors.

① Biological factors: sex, age, weight, height, body mass index, blood pressure, pulse rate, body temperature, overall wellness status, smoking and alcohol consumption history, physical activity, nutritional status, dietary survey, appetite, intake of health supplements, degree of constipation, comorbidities, activities of daily living, physical

resilience, oral health status, gynecological history, medical history, surgical history, medication history and family medical history, physical performance test, range of motion assessment for the knee and lumbar joints, core muscle strength measurement, grip strength measurement, hearing, vision, pulmonary function, pulsometry, brain wave examination, bone density, body composition, clinical laboratory examination (biochemical markers, hormones, hematological markers, urine test), and omics profiles (metabolomics/lipidomics, proteomics).

② Psychological factors: overall wellness status, quality of sleep, quality of life, fear of falling, degree of depression, and cognitive function. Social factors: education level, family relationships, pregnancy and lactation history, overall wellness status, smoking and alcohol consumption history, social relationships, accessibility of health care Services, and quality of life.

③ KM-based phenotype factors: kidney deficiency, five organ deficiency, blood stasis, and core seven-emotions status.

5) Data analysis

We will conduct various analyses within the cohort. The results of the participants' demographic data, the questionnaire-based prevalence of age-related symptoms and KM-based phenotype, physical function, and other outcome variables will be summarized using descriptive summary measures. For continuous outcomes, both univariate and multivariate linear regression analyses will be employed, while logistic regression will be used for dichotomous outcomes. Specifically, group differences (between KM-based phenotypes or between rural and urban areas) will be analyzed using Student's t test and analysis of variance. The relationships between KM-based phenotypes and physical and psychological health, as well as demographic variables, will be analyzed using multiple regression.

Additionally, missing data patterns will be examined, and appropriate methods for handling missing data, such as Last Observation Carried Forward (LOCF) or Multiple Imputation, will be applied. Mixture models will be utilized to identify subgroups of subjects based on their biological and psychological characteristics. To estimate the cumulative risk of outcomes, Kaplan–Meier analysis will be employed. Adjustments for covariates will be made before the follow-up period, during which Cox proportional hazard models will be used to estimate the risk of experiencing any of the outcomes. In addition, prediction modelling will be attempted by incorporating new covariates under investigation. P values less than 0.05 will be considered to indicate statistical significance. All statistical analyses will be conducted using SAS® version 9.4.

Ethical approval

This study received approval from the Institutional Review Board (IRB) of Wonkwang University Korean Medicine Hospital, Iksan, Republic of Korea (approval number: WKUIOMH-IRB-2023-05) on August 16, 2023 and Jangheung Integrative Medical Hospital (approval number: WKUJIM-202307-001) on August 21, 2023. Written informed consent will be obtained from all participants prior to

their enrollment in the study. This study is registered with the Clinical Research Information Service (https://cris.nih.go.kr/cris/en/) (KCT0008863), and the current protocol version is 1.3.

References

1. Statistics Korea. Special estimate of the future population between 2017 and 2067, Daejeon: Statistics Korea; 2019.
2. Beard JR, Officer A, de Carvalho IA, Sadana R, Pot AM, Michel JP, et al. The World report on ageing and health: a policy framework for healthy ageing. Lancet. 2016;387 (10033):2145-2154. doi: 10.1016/S0140-6736(15)00516-4 PMID: 26520231
3. WHO. Decade of healthy ageing 2020–2030, Geneva, Switzerland: World Health Organization; 2020.
4. Cesari M, Sumi Y, Han ZA, Perracini M, Jang H, Briggs A, et al. Implementing care for healthy ageing. BMJ Glob Health. 2022;7(2):e007778. doi: 10.1136/bmjgh-2021-007778 PMID: 35185014
5. Katsagoni CN, Kokkinos P, Sidossis LS. Healthy aging. In: Katsagoni CN, Kokkinos P, Sidossis LS, editors. Prevention and management of cardiovascular and metabolic disease. 2023. p. 91-104. https://doi.org/10.1002/9781119833475.ch7
6. Ronai P, Gallo PM. The short physical performance battery (assessment). ACSM's Health Fit J. 2019;23(6):52-56. doi: 10.1249/FIT.0000000000000519
7. Michel J-P, Ecarnot F. Integrating functional ageing into daily clinical practice. J Frailty Sarcopenia Falls. 2019;4(2):30-35. doi: 10.22540/JFSF-04-030 PMID: 32300715
8. Newman AB. The epidemiology and societal impact of aging-related functional limitations: a looming public health crisis. J Gerontol: A. 2023;78 (Suppl 1):4-7. doi: 10.1093/gerona/glad021 PMID: 37325965
